# Corrigendum

**DOI:** 10.1002/1348-9585.12368

**Published:** 2022-11-01

In Yasuda et al,^1^ the following error was published in Table 2.

**TABLE 2:** Implemented workplace measurements against COVID‐19

Number of workplace measures against COVID‐19					
	0–1	2–3	4–5	≥6	Total
	*n* = 4177	*n* = 3404	*n* = 4603	*n* = 14 852	*N* = 27 036
Refraining from and restriction of business trips	56 (1.3%)	400 (11.8%)	1479 (32.1%)	12 725 (85.7%)	14 660 (54.2%)
Refraining from or restriction on visitors	26 (0.6%)	189 (5.6%)	867 (18.8%)	11 217 (75.5%)	12 299 (45.5%)
Refraining from or requesting a limit on the number of people at social gatherings and dinners	149 (3.6%)	1200 (35.3%)	3340 (72.6%)	14 525 (97.8%)	19 214 (71.1%)
Refraining from or limiting face‐to‐face internal meetings	8 (0.2%)	250 (7.3%)	1418 (30.8%)	13 001 (87.5%)	14 677 (54.3%)
Wearing masks at all times during working hours	551 (13.2%)	2315 (68.0%)	3970 (86.2%)	14 387 (96.9%)	21 223 (78.5%)
Installation of partitions and consideration of workplace layout (e.g. altering desk layout or adjusting flow lines)	83 (2.0%)	748 (22.0%)	2316 (50.3%)	12 587 (84.7%)	15 734 (58.2%)
Recommending workers perform daily temperature checks at their homes	183 (4.4%)	1349 (39.6%)	2892 (62.8%)	12 825 (86.4%)	17 249 (63.8%)
Encouragement of telecommuting	114 (2.7%)	255 (7.5%)	539 (11.7%)	6886 (46.4%)	7794 (28.8%)
Prohibiting eating at a worker's own desk	12 (0.3%)	87 (2.6%)	292 (6.3%)	4206 (28.3%)	4597 (17.0%)
Requesting employees not to come to work when they are not feeling well	279 (6.7%)	1859 (54.6%)	3757 (81.6%)	14 335 (96.5%)	20 230 (74.8%)

**The description of** Table 2 **in the result section:**

“Table 2 shows the number of infection control efforts put in place and the details of each effort. The most commonly implemented infection control measures were “wearing masks at all times during working hours” (79%) and “refraining from or requesting a limit on the number of people at social gatherings and dinners” (71%). In contrast, relatively few companies had implemented “requesting employees not come to work when they are not feeling well” (9%) and “prohibiting workers from eating at their own desk” (17%).”

The text was incorrect and should have read:

“Table 2 shows the number of infection control efforts put in place and the details of each effort.

The most commonly implemented infection control measures were “wearing masks at all times during working hours” (79%) and “requesting employees not to come to work when they are not feeling well” (75%). In contrast, relatively few companies had implemented “prohibiting workers from eating at their own desk” (17%).”

The corrected Table 2 is below:

The authors apologize for this error.
